# Evidence for decreased interaction and improved carotenoid bioavailability by sequential delivery of a supplement

**DOI:** 10.1002/fsn3.409

**Published:** 2016-07-28

**Authors:** Dawna Salter‐Venzon, Valentina Kazlova, Samantha Izzy Ford, Janjira Intra, Allison E. Klosner, Kevin W. Gellenbeck

**Affiliations:** ^1^ Amway R&DNutrilite Health InstituteBuena ParkCAUSA

**Keywords:** Bioavailability, carotenoids, digestion, nutrition, sequential release

## Abstract

Despite the notable health benefits of carotenoids for human health, the majority of human diets worldwide are repeatedly shown to be inadequate in intake of carotenoid‐rich fruits and vegetables, according to current health recommendations. To address this deficit, strategies designed to increase dietary intakes and subsequent plasma levels of carotenoids are warranted. When mixed carotenoids are delivered into the intestinal tract simultaneously, competition occurs for micelle formation and absorption, affecting carotenoid bioavailability. Previously, we tested the *in vitro* viability of a carotenoid mix designed to deliver individual carotenoids sequentially spaced from one another over the 6 hr transit time of the human upper gastrointestinal system. We hypothesized that temporally and spatially separating the individual carotenoids would reduce competition for micelle formation, improve uptake, and maximize efficacy. Here, we test this hypothesis in a double‐blind, repeated‐measure, cross‐over human study with 12 subjects by comparing the change of plasma carotenoid levels for 8 hr after oral doses of a sequentially spaced carotenoid mix, to a matched mix without sequential spacing. We find the carotenoid change from baseline, measured as area under the curve, is increased following consumption of the sequentially spaced mix compared to concomitant carotenoids delivery. These results demonstrate reduced interaction and regulation between the sequentially spaced carotenoids, suggesting improved bioavailability from a novel sequentially spaced carotenoid mix.

## 1 Introduction

Carotenoids, the pigments responsible for lending color to various fruits and vegetables, are considered beneficial substances in human health. The results of epidemiological studies over the past 30 years consistently demonstrate that higher levels of total plasma carotenoids are associated with improved health outcomes (Hak et al., 2004; Hozawa et al., 2006; Wu et al., 2004). Also, an inverse relationship can be seen between the intake of carotenoid‐rich foods and the risk for chronic disease (Krinsky & Johnson, 2005; Mayne, 1996).

One of the most well‐studied activities is with provitamin A activity as the human body can produce essential vitamin A from certain carotenoids, notably β‐ and α‐carotene. Also, there is substantial research showing the antioxidant potential of carotenoids and their protective effects on various tissues against chronic conditions, including: cataract formation, coronary heart disease, stroke, enhancement of the immune system, protection from sunburn, inhibiting the development of certain types of cancer and possibly even antiobesity effects (Arnlov et al., 2009; Benedich, 1989; Eye Disease Case‐Control Study Group, 1993; Giovannucci et al., 1995; Hak et al., 2004; Hozawa et al., 2006; Maiani et al., 2009; Markovits, 2009; Ribaya‐Mercado & Blumberg, 2004; Stahl & Sies, 2002; van Poppel, 1993).

Despite the notable health benefits, the majority of human diets worldwide are repeatedly shown to be inadequate in intake of carotenoid‐rich fruits and vegetables, according to current health recommendations (Blanck, Gillespie, Kimmons, Seymour, & Serdula, 2008; Murphy, Barraj, Spungen, Herman, & Randolph, 2014; Murphy et al., 2012; Tennant, Davidson, & Day, 2014; WHO, 2002). One strategy to improve the variety and quantity of carotenoids included in dietary regimens is through fortification or supplementation. Consumption of a mixed variety of carotenoids is suggested to be more beneficial than focusing on supplementation of a single carotenoid in lieu of others (Astley, Ruan, Archer, & Southon, 2004; Liu, 2003; Tapiero, Townsend, & Tew, 2004). In addition to increased dietary carotenoid consumption, a mixed carotenoid supplement should deliver a diversity of carotenoids contained within healthy diets comprised of adequate fruit and vegetable content.

When considering the formulation of a mixed carotenoid supplement, absorption and bioavailability are the key variables influencing efficacy. The small intestine is responsible for absorbing hydrophobic substances, such as carotenoids, so they can be subsequently delivered through the blood stream to peripheral tissues. During digestion in the upper intestine, carotenoids are incorporated into mixed micelles composed of phospholipids, lipids, cholesterol, and bile salts and absorbed into the enterocyte through passive and facilitated diffusion (Hollander & Ruble, 1978; Valacchi, Sticozzi, Lim, & Pecorelli, 2011; Van Bennekum et al., 2005; Yonekura & Nagao, 2007). After absorption, carotenoids are packaged into plasma and secreted into the lymph system for transport to the bloodstream.

However, when various mixed carotenoids are delivered into the intestinal tract together at higher concentrations, the compounds compete for micelle formation and subsequent intestinal absorption (Maiani et al., 2009; van den Berg, 1998, 1999; van het Hof, West, & Westerate, 2000). Carotenoids may also compete with one another for uptake and metabolism in the enterocyte and for incorporation into the plasma (Kostic, White, & Olsen, 1995; Paetau, Huiping, Goh, & White, 1997; van den Berg & Van Vliet, 1998). This competitive interaction can hinder carotenoid absorption and transportation to target end sites.

In this regard, a method delivering mixed carotenoids that are temporally and spatially separated within the gastrointestinal tract may minimize micelle competition, consequently improving overall bioavailability of individual carotenoids. The hypothesis tested here is that a mix utilizing carotenoid spacing will have better absorption due to decreased competition among the individual carotenoids for improved bioavailability and uptake.

In this study, a mix of natural carotenoids was tested incorporating a sequential release of individual carotenoids across gut transit time of approximately 6 hr to elucidate the impact on uptake.

The designed carotenoid mix is composed of the primary carotenoids found in human plasma that are associated with a variety of fruit and vegetable intake: β‐ and α‐ carotene, lutein, lycopene, and zeaxanthin (Maiani et al., 2009). In light of its reported health benefits but general exclusion in the average diet, astaxanthin was also included in the mix to determine if competitive interaction exists with the common dietary carotenoids. (Fassett & Coombes, 2012; Yuang, Peng, Jin, & Wang, 2011).

The mix of carotenoids were included in a set ratio, designed by incorporating key aspects of well‐accepted dietary guidelines and considering typical and wide‐spread dietary inadequacies, such as those exposed by other recent results (Murphy et al., 2012, 2014). These results suggested a carotenoid intake amount that would be found in a “prudent diet” following the World Health Organization's recommendation of at least five servings of fruits and vegetables per day (Joint FAO/WHO Workshop on Fruit and Vegetables for Health, 2004). The amount and proportion of carotenoids found in this “prudent diet” served as the baseline to create a ratio of carotenoids that, when consumed, would help improve carotenoid intake. Notably, the ratio was not designed to simply increase the amounts of carotenoids but rather to promote a mix of carotenoids as would be found in a healthy diet with a minimum of five servings of fruit and vegetables (Blanck et al., 2008; Gellenbeck, Salter‐Venzon, Lala, & Chavan, 2012; Murphy et al., 2012). This ratio is shown in Table 1.

**Table 1:** Carotenoids and their release profile in experimental dosage

Carotenoid and source	CCM Beadlet composition (mg carotenoid/g beadlet)	CCM 1‐ time dose (mg carotenoid)	SSCM Beadlet composition (mg carotenoid/g beadlet	SSCM 1‐ time dose (mg carotenoid)	Release profile (hr)
β‐carotene from *Dunaliella* algae & palm fruit	26.0	36.0	23.9	36.0	4–6
α‐carotene from palm fruit	5.3	7.3	4.2	7.7	4–6
Lycopene from tomato	8.0	11.1	7.7	14.1	3–4
Lutein from marigold	14.1	19.4	12.5	19.8	2–3
zeaxanthin from marigold	2.7	3.7	2.9	4.4	2–3
Astaxanthin from *Haemaotcoccus* algae	2.2	3	2.2	3	1–2

CCM, concomitant carotenoid mix; SSCM, sequentially spaced carotenoid mix.

The designed spacing sequence was informed by the interactions between specific carotenoids in addition to evidence of when each carotenoid might first appear in the triacylglycerol‐rich fraction of plasma after oral administration in order to approximate when peak absorption might occur (Bierer, Merchen, & Erdman, 1995; Cardinault et al., 2003; Johnson, Qin, Krinsky, & Russell, 1997; O'Neill & Thurnham, 1998; Tysandier et al., 2002; van den Berg, 1999; Van het Hof et al., 2000; Zaripheh & Erdman, 2002). Previously, a carotenoid mix was designed and tested *in vitro* for its ability to deliver individual carotenoids sequentially spaced from one another over the 4 to 6 hr of transit time through the upper intestinal system of the human gut (Gellenbeck et al., 2012). This study expands on the previous work by investigating the change of plasma carotenoid concentrations in adult humans after oral consumption of a mix of carotenoids that are sequentially spaced from one another, in comparison to a matched‐dose mix of carotenoids that are delivered to the intestinal system concomitantly. The aim of this study was to determine if separating individual carotenoids spatially and temporally throughout the transit time of the upper gastrointestinal tract lends absorption and bioavailability benefits over a concomitantly delivered carotenoid mix.

## 2 Materials and Methods

Twelve healthy, nonsmoking men and women volunteers between the ages of 18 and 60 were recruited for this study. Prior to entry, written consent was obtained from each volunteer. All procedures adopted were in accordance with the Helsinki Declaration and this study was approved by Alpha Independent Review Board (San Clemente, CA). The volunteers were judged to be in good health, on the basis of a medical exam, with no history of chronic disease, blood cholesterol levels between 120 and 219 mg/dl, triglycerides between 35 and 164 mg/dl, body mass index between 20 and 28 kg/m^2^, and not taking any dietary supplements in the 2 months prior to the study. The volunteers were instructed to maintain their normal diet and exercise habits throughout the duration of the study, with the exception of avoiding consumption of carotenoid‐rich fruit or vegetables in the 3 days prior to each visit. Subjects were verbally instructed on carotenoid‐rich foods and given a written list of foods to avoid during this time frame. Volunteers arrived at the testing center on the day of experiment following a 12‐hr overnight fast. Compliance was verified through a three‐day diet history recall. Each subject was randomly assigned to one of two matched‐dose, but differently delivered, mixes of carotenoid beadlets in the form of a two‐piece capsule containing noncompressed powder (as opposed to a compressed tablet or soft gel delivery format): one in which the individual carotenoids are sequentially separated from one another (sequentially spaced carotenoid mix, SSCM) or one in which each carotenoid is delivered concomitantly (concomitant carotenoid mix, CCM). The mix of carotenoids were consumed 20 min after eating a carotenoid‐free, high‐fat meal (≈50% of calories from fat). The meal, composed of white biscuits, white gravy, hash browns, white bread, coffee and/or water, contained 1270 calories and 68 grams of dietary fat. A baseline fasting blood sample was taken (time 0), followed by an allotted 20 min for the consumption of the carotenoid‐free meal, and additional blood samples taken 1, 2, 4, 6, and 8 hr after consumption of the carotenoid mix. Subjects did not receive a second meal, or any other food or drink, other than water, for the remaining 8 hr of the postprandial experiment.

Fourteen to sixteen days later, the subjects returned to the research center to repeat the procedure in a double‐blinded, repeated‐measure, crossed‐over design such that each subject received the opposite matched‐dose carotenoid mix from the previous visit. It is known that individual subjects can absorb carotenoids differently in dynamics and magnitude, leading to large intersubject variability despite the study being well‐controlled (Brown et al., 1989; O'Neill & Thurnham, 1989). Commonly, these differences are noted by referring to individuals as being either strong or weak responders, and indicates that carotenoid responses are, at least in part, under the control of specific genetic variants (Wang, Edwards, & Clevidence, 2013). However, while there can be large intersubject variability in controlled studies, particular individuals tend to respond to a specific carotenoid dose in a rather constant manner, even if the dose is separated by a matter of months. (O'Neill & Thurnham, 1989). The repeated measures experimental design used here attempted to minimize the potential effects of interindividual heterogeneity and improve statistical strength with fewer subjects (twelve was considered adequate), as each subject's response was compared across carotenoid format.

### 2.1 Carotenoid mix composition

All active ingredients contained within the carotenoid mixes were derived from natural sources and combined with standard inert ingredients to form the carotenoid mixes as described previously (Gellenbeck et al., 2012). Briefly, the carotenoid mix to include sequential spacing was prepared by Omniactive Health Technologies (Morristown, NJ) using proprietary technology. Layers of carotenoid extracts are applied to an inner sugar core along with functional release coats and an outer protective coat. The final form is a small beadlet suitable for inclusion in functional foods, capsules, or tablets. In addition, a matched carotenoid mix was prepared without the sequential spacing for use as a comparator. The active ingredient sources are as follows: Natural β‐carotene derived from *Dunaliella* algae and palm fruit, natural α‐carotene derived from palm fruit, lutein from marigold flowers, lycopene from tomato, and astaxanthin from *Haemotococcus* algae. The amounts contained in SSCM and CCM could be closely, but not perfectly, matched due to the nature of the methods used to sequentially space the carotenoids in SSCM. Therefore, β‐carotene and astaxanthin were chosen as the carotenoids used as reference to match the dosage provided from each formulation, that is, β‐carotene and astaxanthin doses were exactly the same in both formulations and the small differences in the other carotenoids were taken into account during analysis. The compostion, ratio, and amount of mixed carotenoids provided to subjects is shown in Table 1. The dose of carotenoids used for this experiment was selected from unpublished pilot data. In these pilot experiments, four volunteer subjects were provided different amounts of carotenoids to establish the threshold at which plasma carotenoid levels could be detected and measured. This information served as the basis for the amount of carotenoids to be used in this experiment. While this dosage is higher than would be recommended on a daily basis, this was a one‐time dose intended to accentuate competitive interactions.This level is not above single‐dosages used in other kinetic carotenoid studies and there are no established dietary upper limits for these carotenoids (Johnson et al., 1997; Maiani et al., 2009; O'Neill & Thurnham, 1989).

### 2.2 Plasma preparation and carotenoid analysis

Blood was collected in EDTA‐treated evacuated tubes and plasma was prepared immediately by centrifugation, stored overnight at −20°C and shipped under dry ice to Craft Technologies, Inc. (Wilson, NC) for carotenoid quantification at each time point.

The compounds were identified and quantified using high‐performance liquid chromatography (HPLC). For plasma extraction and HPLC methods, a modification of the procedures described by Nomura, Stemmermann, Lee, & Craft, (1997) was used. The method variability is 3–10% with the 10% being for minor components such as alpha‐carotene, alpha‐cryptoxanthin, and zeaxanthin. The method has performed well in the NIST QA program measuring blinded samples, typically within one SD of the assigned value.

### 2.3 Statistical analysis

Data are expressed as means ± standard error of the means (SEMs). To decrease heterogeneity of responses, postprandial plasma carotenoid concentrations are expressed as incremental responses (fasting baseline values set to equal zero). The areas under the curve (AUC) of the postprandial plasma responses of each carotenoid were calculated by trapezoidal approximation after subtracting baseline concentration. AUCs obtained for SSCM and CCM were compared using a paired *t* test (appropriate for a repeated measures design) and differences associated with a *p *≤ 0.05 were regarded as statistically significant.

To further assess the interactions between individual carotenoids, a Spearman correlation was performed on the raw concentration SSCM and CCM values. Spearman's Correlation was used to visualize the association between carotenoid concentrations. Co‐regulation correlations were clustered hierarchically using the Ward method. All statistical comparisons were performed using the statistical analysis software JMP (SAS Institute, Cary, NC).

## 3 Results

Study Population: Subject baseline characteristics are presented in Table 2. Of the 12 subjects enrolled, four were male and eight were female. Ages were between 21 and 58 (mean 38.4), body mass index was between 21.3 and 27.8 kg/m^2^, and plasma cholesterol and triglyceride levels were within acceptable ranges. Table 3 illustrates no significant differences in baseline plasma carotenoid concentrations between experimental days, prior to the subjects consuming either SSCM or CCM. In addition, there was no significant difference between total carotenoid concentrations at baseline between experimental conditions (CCM 738 ng/ml vs. SSCM 688 ng/ml, *p* = .274). Further analysis showed no significant effects of blood lipids, body weight, body mass index, age, or gender on pre‐, post‐prandial, or incremental change of plasma carotenoid concentrations.

**Table 2:** Study Baseline Characteristics. All triglycerides and cholesterol levels were within acceptable ranges

	*N*	Mean	Std Err	Std Dev	Min	Max
Age	12	38.42	2.88	9.98	21	58
BMI	12	24.90	0.65	2.26	21.3	27.8
Total Cholesterol	12	173.92	9.74	33.74	126	221
TRIG1 ‐ screening “fasting”, mg/dL	12	74.08	7.19	24.92	46	140
Gender						
F	8					
M	4					

CCM, concomitant carotenoid mix; SSCM, sequentially spaced carotenoid mix.

**Table 3:** Baseline plasma carotenoid concentrations of subjects. Baseline comparison (*p*‐values are provided for two‐sided paired *t* test). α = .05 level of significance baseline values for each carotenoid and also total carotenoids did not differ significantly between CCM and SSCM groups

Carotenoid	Group	Mean concentration, ng/ml	Mean difference, ng/ml	Std Err	*p*‐value
Total lycopene, mcg/ml	CCM	258.3	−41.6	0.01986	.0602
	SSCM	216.8			
Alpha‐carotene, mcg/ml	CCM	46.0	57.5	0.00616	.3709
	SSCM	51.8			
Total beta‐carotene, mcg/ml	CCM	184.7	65.8	0.01768	.7167
	SSCM	191.3			
Astaxanthin, mcg/ml	CCM	11.9	–1.3	0.00105	.2600
	SSCM	10.7			
Total Lutein, mcg/ml	CCM	177.3	−14.0	0.00995	.1869
	SSCM	163.3			
Total Zeaxanthin, mcg/ml	CCM	60.1	−5.6	0.00355	.1438
	SSCM	54.5			
Total carotenoids, mcg/ml	CCM	738.3	−50.1	0.04351	.2741
	SSCM	688.2			

CCM, concomitant carotenoid mix; SSCM, sequentially spaced carotenoid mix.

### 3.1 Effect of spacing carotenoids on postprandial plasma response

**Figure 1:**
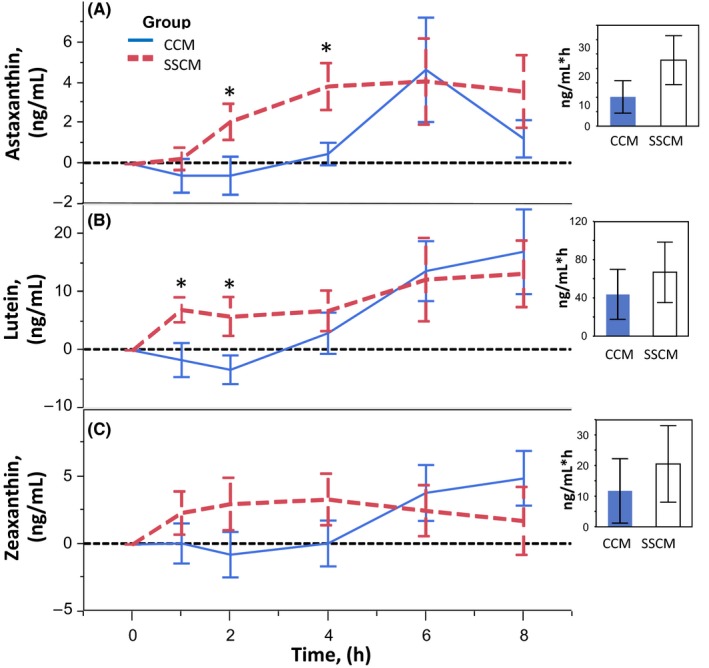
Effect of delivery on postprandial plasma carotenoid response—Astaxanthin, lutein, zeaxanthin. Error bars are constructed using one standard error from the mean. Two‐sided paired *t* test illustrates astaxanthin response is significantly higher at time points 2 and 4, and lutein at 1 and 2 hr after SSCM than after CCM. Overall incremental areas under the curve (AUC) is not significantly different between delivery types. α=.05 level of significance. (CCM, concomitant carotenoid mix; SSCM, sequentially spaced carotenoid mix)

**Figure 2:**
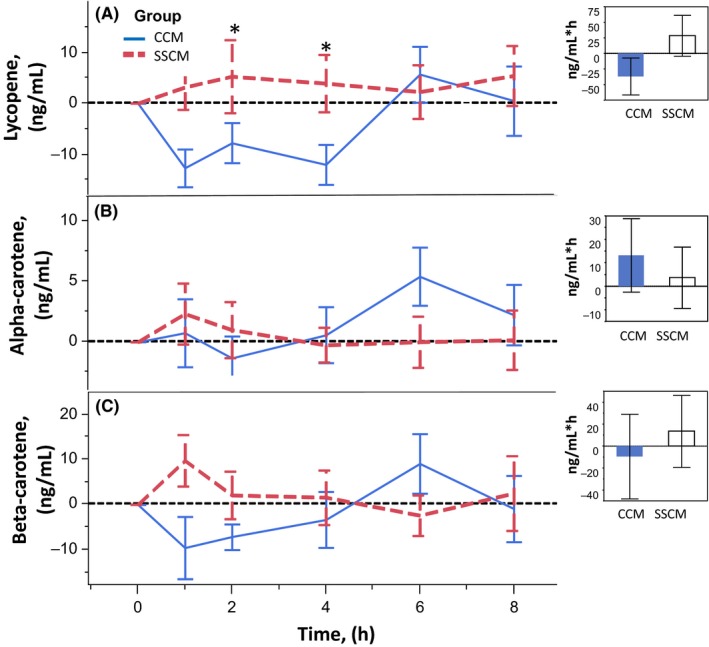
Effect of delivery on postprandial plasma carotenoid response—Lycopene, Alpha‐carotene, Beta‐carotene. Error bars are constructed using one standard error from the mean. Two‐sided paired *t* test illustrates lycopene response after SSMC is significantly higher at time points 2 and 4 hr than after CMC. Overall incremental areas under the curve (AUC) is not significantly different between delivery types, α=.05 level of significance

The changes in individual postprandial plasma carotenoid concentrations after either CCM or SSCM are shown in Figures 1 and 2. Figure 1 illustrates the astaxanthin, lutein, and zeaxanthin plasma responses. Astaxanthin response to SSCM begins 1 hr following consumption, peaks at 4 hr, and plateaus for the remainder of the experimental measures. In contrast, after consumption of CCM, the same subjects demonstrate a decrease in astaxanthin for the first 2 hr, which reverses to peak by 6 hr, then quickly plummets over the remaining measured time points. Astaxanthin measures were significantly higher after SSCM than CCM at 2 and 4 hr, however, the statistical difference in incremental change did not remain over 8 hr. The SSCM AUC was 12.75 ng/ml*hr ± 15.7 greater than CCM AUC (*p* = .157). Lutein and zeaxanthin show similar plasma responses over 8 hr after consumption of SSCM and CCM, which is not surprising since these two carotenoids have similar chemical structure (Bierer et al., 1995; Kotake‐Nara & Nagao, 2011; Nagao, 2011). SSCM showed a higher response in these two compounds during the first 4 hr, with lutein showing a significantly higher response at the first two time points. However, the incremental changes of both of these carotenoids over the 8 hr experiment were not significantly different. Lutein and zeaxanthin mean incremental response to SSCM over CCM was 23.25 ng/ml*hr ± 33.1(*p* = .249) and 8.83 ng/ml*hr ± 17.5 (*p* = .312), respectively.

Figure 2 illustrates the lycopene, β‐ and α‐ carotene plasma responses following consumption of SSCM or CCM. Lycopene responses after SSCM showed a somewhat small elevation at 1 hr that largely plateaued, but in the last 2 hr of experimental measures appeared to be rising. Interestingly, CCM postprandial plasma lycopene responses showed a measurable decrease over the first 4 hr of the experiment, a quick peak at 6 hr, followed by a decline at the final 8 hr measure. Lycopene responses were significantly higher for the first 4 hr following SSCM consumption and overall difference in AUC was trending but not statistically different (*p* = .056) between the two carotenoid formats. SSCM mean incremental AUC was 65.42 ng/ml*hr greater than CCM mean incremental AUC for lycopene. The plasma response curves following CCM for both α‐ and β‐carotene were somewhat similar to each other. Both demonstrated a decrease from baseline for about 4 hr, a peak at 6 hr, followed by a decline toward baseline by the final measurement at 8 hr. Although the curve dynamics appear differently shaped, there were no statistically significant differences at any time points between the two carotenoid mixes, and the overall mean incremental AUCs also were not significantly different for either β‐ and α‐ carotene. The mean incremental AUC for α‐ carotene was 9.5 ng/ml* hr ± 24.8 less after SSCM consumption than CCM consumption (*p* = .645), while that for β‐carotene was 23.0 ng/ml*hr ± 61.2 greater after SSCM than after CCM (*p* = .357). Following consumption of SSCM, the incremental AUC of α‐ and β‐ carotene did not demonstrate much change over the course of the measured time points. The predominate difference between SSCM and CCM regarding β‐ and α‐ carotene was that the drop below baseline seen after CCM was not apparent after SSCM. Additionally, CCM appeared to peak at 6 hr while response of α‐ and β‐ carotene following SSCM was relatively flat. The final measurement (8 hr) of SSCM appeared to be that of an increasing response, so perhaps the peak of plasma carotenoid change was later than our experiment was designed to measure, suggesting that a longer experimental period would be appropriate. An additional variable would be the inclusion of a secondary meal that could affect the uptake of carotenoids from the initial experimental dosage.

**Figure 3:**
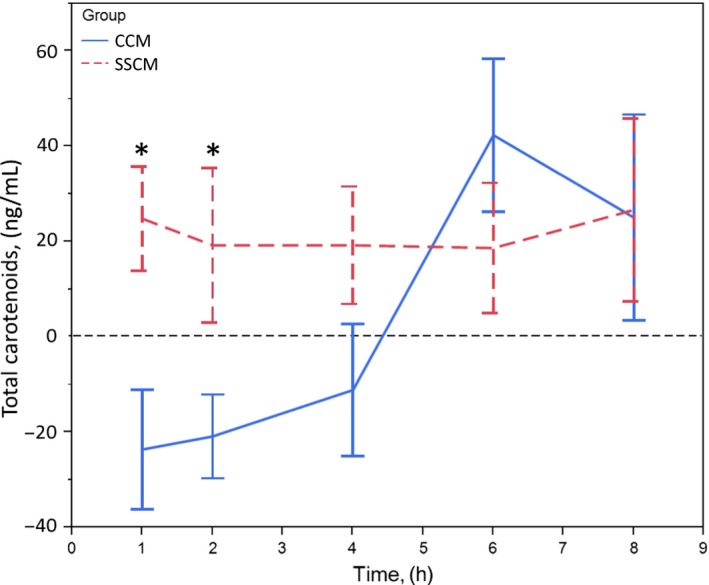
Effect of delivery on postprandial plasma response—total carotenoids. Error bars are constructed using 1 standard error from the mean. Two‐sided paired *t* test illustrates total carotenoid response after SSCM is significantly higher at 1 and 2 hr than after concomitant carotenoid mix (CCM). Note the large dip from baseline carotenoid values for the first 4–5 hr following CCM delivery that may be indicative of increased competition among the individual carotenoids for intestinal absorption. Overall incremental areas under the curve (AUC) is not significantly different between delivery types, α=.05 level of significance

The sum of the mean incremental plasma responses of all carotenoids following consumption of SSCM or CCM is shown in Figure 3. The postprandial plasma responses demonstrate a significant difference in the total carotenoid content at hours one–three between SSCM and CCM. Interestingly, the mean incremental plasma response showed a marked decrease from baseline following consumption of CCM for the first 4 hr, a peak level at 6 hr, followed by a steep decline. In contrast, the plasma carotenoid response following SSCM shows a steady level throughout the experimental time points. Overall, the mean difference in incremental AUC between SSCM and CCM was 123.75 ng/ml*hr ± 136.7. The difference was in favor of SSCM, but was not statistically significant at *p* = .193, most likely due to the large variability inherent in these type of small‐scale experiments with carotenoids.

### 3.2 Interactions and co‐regulations of plasma carotenoid responses

**Figure 4:**
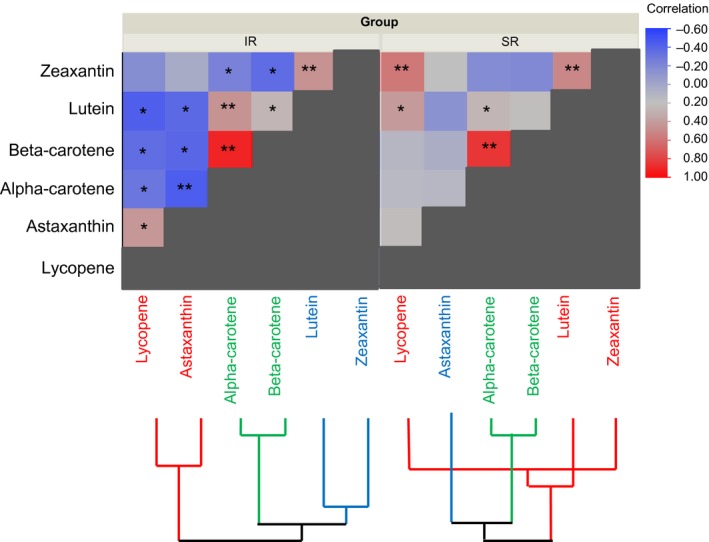
Associations among carotenoid concentrations in plasma. Heat map displaying Spearman correlation coefficients, asterisks indicate magnitude of corresponding *p*‐values (*p < .05, **p < .0001). Hierarchical clustering was built using Ward method. Scale on the right illustrates intensity of color indicating greater correlations—blue as a negative correlation and red as a positive correlation. After concomitant delivery of mixed carotenoids (CCM, left) the interactions between individual carotenoids are high, indicating a high degree of negative association. After a sequentially spaced delivery of mixed carotenoids (SSCM, right), the interactions are much less apparent or absent. Hierarchical clustering also shows a much different picture of association between delivery types

Raw concentration values (original data, not transformed) were assessed using nonparametric Spearman's correlation ranking for each carotenoid from SSCM and CCM and compared to one another. Figure 4 demonstrates a heat map of the Spearman correlation with hierarchical clustering. The scale is represented by intensity of color with blue being a negative correlation, red being positive, and pale colors indicating weak or absence of correlation. The intensely colored boxes on the CCM heat map (Fig. 4, left) illustrate the postprandial plasma responses of individual carotenoids that are more strongly correlated with one another, with 15 Spearman correlation coefficients (Table 4) found from the plasma responses following CCM consumption. Of these 15 coefficients, nine were identified with powerful negative correlations such as astaxanthin with α‐ carotene (−0.449, *p* < .0001), astaxanthin with β‐carotene (−0.396, *p* = .0006), and lycopene with lutein (−0.431, *p* = .0001), supporting the well‐known notion that mixed carotenoids inhibit one another in absorption and early metabolism dynamics (Maiani et al., 2009; van den Berg, 1998, 1999; Van het Hof et al., 2000). In comparison, the muted color scheme apparent on the Spearman's heat map for SSCM (Fig. 4, right) illustrates that plasma carotenoid responses are less affected by one another. Carotenoid co‐regulation following SSCM consumption is also less likely to be inhibitory than after CCM, as only three Spearman correlation coefficients were mildly negative with this type of carotenoid delivery; astaxanthin with lutein (−0.140, *p* = .2413), α‐ carotene with zeaxanthin (−0.176, *p* = .1397), and β‐carotene with zeaxanthin (−0.195, *p* = .1015).

**Table 4:** Spearman's correlation coefficients

Variable	By variable	CCM		SSCM	
		Spearman Rho	Prob>|Rho|	Spearman Rho	Prob>|Rho|
Alpha‐carotene, mcg/ml	Lycopene, mcg/ml	−.3077	.0086*	.1391	.2441
Beta‐carotene, mcg/ml	Lycopene, mcg/ml	−.3586	.0020*	.1175	.3255
Beta‐carotene, mcg/ml	Alpha‐Carotene, mcg/ml	.9151	<.0001*	.8541	<.0001*
Astaxanthin, mcg/ml	Lycopene, mcg/ml	.4344	.0001*	.225	.0574
Astaxanthin, mcg/ml	Alpha‐Carotene, mcg/ml	−.4496	<.0001*	.1283	.2827
Astaxanthin, mcg/ml	Beta‐Carotene, mcg/ml	−.3957	.0006*	.0525	.6617
Lutein, mcg/ml	Lycopene, mcg/ml	−.4361	.0001*	.4189	.0002*
Lutein, mcg/ml	Alpha‐Carotene, mcg/ml	.4574	<.0001*	.271	.0213*
Lutein, mcg/ml	Beta‐Carotene, mcg/ml	.2716	.0210*	.2222	.0606
Lutein, mcg/ml	Astaxanthin, mcg/ml	−.3799	.0010*	−.1399	.2413
Zeaxanthin, mcg/ml	Lycopene, mcg/ml	−.1653	.1651	.5828	<.0001*
Zeaxanthin, mcg/ml	Alpha‐Carotene, mcg/ml	−.2384	.0437*	−.1758	.1397
Zeaxanthin, mcg/ml	Beta‐Carotene, mcg/ml	−.3667	.0015*	−.1945	.1015
Zeaxanthin, mcg/ml	Astaxanthin, mcg/ml	.032	.7898	.1943	.1019
Zeaxanthin, mcg/ml	Lutein, mcg/ml	.4601	<.0001*	.5141	<.0001*

CCM, concomitant carotenoid mix; SSCM, sequentially spaced carotenoid mix. Asterisks indicate magnitude of corresponding *p*‐values (*p < .05, **p < .0001).

## 4 Discussion

The aim of this study was to determine if a spatially and temporally separated carotenoid mix would lend absorption and bioavailability benefits over a concomitantly delivered carotenoid mix. The hypothesis states that while both are matched in carotenoid content, the mix utilizing carotenoid spacing would have better absorption due to decreased competition among the individual carotenoids for uptake and metabolic enzymes.

The results indicate that a mix composed of naturally sourced carotenoids is taken up into the plasma after consumption by human volunteers. Although both SSCM and CCM elicited measurable increases in postprandial plasma carotenoid responses after consumption, the response curves between the two forms were noticeably different in both shape and magnitude in the same subject.

There were differences in curve shapes and peak timing of the postprandial plasma responses following SSCM in comparison to CCM. The CCM resulted in a plasma response that, in general, appeared to peak at the 6 hr measurement, followed by a quick decline toward baseline by the end of the experiment. The key significant differences between the two mixes are shown at the early time points. SSCM resulted in a significantly higher plasma response in the first 5 hr following consumption, primarily at hours one through four with astaxanthin, lutein, lycopene, and α‐carotene, (Figs. 1 and 2) supporting the notion that sequentially spacing a mix of carotenoids could be a favorable method to improve dietary carotenoid absorption. Despite the significance at early time points, analysis of AUC of all carotenoids over the entire study duration (Fig. 3) does not reach statistical significance (*p* = .193). However, the increased total AUC for lycopene trended toward significance (*p* = .056).

Further support of a decreased interaction among individual carotenoids for absorption following the SSCM comes from the results of the nonparametric Spearman's correlation ranking analysis. In agreement with previous findings, this analysis provides evidence that individual carotenoids released into the gut lumen all at one time are highly interactive and tend to inhibit the uptake of one another (Kostic et al., 1995; Maiani et al., 2009; Paetau et al., 1997; van den Berg, 1998, 1999; van den Berg & Van Vliet, 1998; Van het Hof et al., 2000). In contrast, following SSCM, the results show a decreased interactivity and co‐regulation among individual carotenoids, presumably due to decreased uptake competition.

Because there is natural variation in an individual's ability to absorb carotenoids, large intersubject variability is a frequent issue even in well‐controlled studies (Brown et al., 1989; Maiani et al., 2009; O'Neill & Thurnham, 1998). In this study, the intersubject variability was mitigated using a cross‐over design where each subject served as its own comparator and incremental changes were evaluated from their baseline values. However, some variability in our study remained as evidenced by a larger standard error accompanying a few of the measures, underpowering its ability to achieve statistical significance. The data from this work can be used to sufficiently power a future study with a larger number of subjects.

The total postprandial incremental AUC for β‐ and α‐carotene following SSCM was flatter than expected. Individual analysis of each subject's β‐ and α‐carotene response did not elucidate any clear nonresponders. Thus, the flat response following SSCM could be due to these two carotenoids not releasing as well in the human gut as in the previously conducted in vitro lab simulations (Gellenbeck et al., 2012), or that the timeline for measurements in this study was too short to capture the peak of the postprandial plasma response to β‐ and α‐carotene. Investigation of the postprandial β‐carotene plasma or the chylomicron fraction of plasma, the fraction that represents the first uptake from dietary sources, illustrates that peak response typically occurs 4–5 hr after consumption of this carotenoid (Bierer et al., 1995; Johnson et al., 1997; O'Neill & Thurnham, 1998; Paetau et al., 1997; Traber, Diamond, Lane, Brody, & Kayden, 1994; Tysandier et al., 2002; van den Berg & Van Vliet, 1998). Since the β‐ and α‐carotene in the SSCM were spaced toward the end of the 6 hr time, the peak response may occur later than what was captured in the 8 hr experiment period, up to 10–13 hr after consumption. A follow‐up study incorporating more subjects, focusing on the chylomicron fraction, and using longer measurement times that would allow a return of plasma concentrations to baseline is warranted to help answer this question.

The plasma carotenoid response following CCM consumption demonstrated a curious and relatively consistent decrease from baseline for the first 4 hr. A similar phenomenon has been noted in a few examples measuring β‐carotene as well, following a mixed carotenoid or a higher dose (Johnson et al., 1997; Paetau et al., 1997), but the cause of this drop is not obvious. Movement across the mucosal cells of the intestinal wall has long been thought to take place through passive diffusion (El‐Gorab, Underwood, & Loerch, 1975; Hollander & Ruble, 1978). However, recent lines of investigation show the presence of facilitated diffusion for the absorption and subsequent early metabolism of carotenoids (Valacchi et al., 2011; Van Bennekum et al., 2005). Therefore, in the case of CCM, the decrease in the carotenoid response from baseline values may be due to competition among the carotenoids, competition from facilitated diffusion proteins, or perhaps carotenoid efflux back to the lumen (Nagao, 2011). Because of the more dispersed release of carotenoids across time and the length of the upper intestinal tract, this competition effect may be mitigated following SSCM. Directed experimentation is required to clarify these observations.

In summary, the results obtained show that mixed carotenoids are bioavailable and result in a plasma carotenoid response after consumption in healthy volunteers. However, when the mixed carotenoids are delivered in a format that releases carotenoids sequentially across the gut lumen, competition for absorption appears to be lessened in comparison to a format that releases its contents at one time. In general, the postprandial plasma response to a sequentially spaced carotenoid mix increased uptake, especially during the first hours following consumption. Further investigation using a larger subject group and a longer experimental time line is warranted.

## Funding Information

This work was funded by Access Business Group, LLC.

## Conflict of Interest

All the authors are employees of Access Business Group International, LLC.
